# Multiple heteroresistance to tigecycline and colistin in *Acinetobacter baumannii* isolates and its implications for combined antibiotic treatment

**DOI:** 10.1186/s12929-023-00914-6

**Published:** 2023-06-07

**Authors:** Jeongwoo Jo, Ki Tae Kwon, Kwan Soo Ko

**Affiliations:** 1 Department of Microbiology, Sungkyunkwan University School of Medicine, Suwon, 16419 Republic of Korea; 2 Department of Internal Medicine, School of Medicine, Kyungpook National University, Daegu, Republic of Korea

**Keywords:** *Acinetobacter baumannii*, Tigecycline, Colistin, Multiple heteroresistance, Combination therapy

## Abstract

**Background:**

We investigated the presence of heteroresistance against both tigecycline and colistin in *Acinetobacter baumannii* and then evaluated the effectiveness of combined antibiotic treatment given the existence of discrete tigecycline- and colistin-resistant subpopulations.

**Methods:**

We performed population analysis profiling (PAP) to evaluate the degree of composite heteroresistance in *A. baumannii* isolates, with the extent of this resistance quantified using subsequent antibiotic susceptibility testing. We then evaluated the amino acid sequence of PmrBAC and the relative mRNA expression levels of *pmrB.* Finally, we investigated the combined antibiotic efficacy of tigecycline and colistin in multiple-heteroresistant isolates using dual PAP and in vitro time-killing assays.

**Results:**

All tigecycline-heteroresistant *A. baumannii* isolates, with the exception of one colistin-resistant isolate, were also heteroresistant to colistin. Evaluations of the colistin-resistant subpopulations revealed amino acid alterations in PmrA and PmrB and increased expression of *pmrB*. All tigecycline-resistant subpopulations were susceptible to colistin, and all colistin-resistant subpopulations were susceptible to tigecycline. Dual PAP analysis using tigecycline and colistin showed no heteroresistance, and in vitro time-killing assays revealed that a combination of these two antibiotics effectively eliminated the bacterial cells.

**Conclusion:**

Our results suggest that multiple heteroresistance to tigecycline and colistin is highly prevalent among *A. baumannii* clinical isolates and that these resistant subpopulations exist independently in single multiple heteroresistant isolates. Therefore, our findings may explain the success of combined antibiotic therapies in these infections.

**Supplementary Information:**

The online version contains supplementary material available at 10.1186/s12929-023-00914-6.

## Introduction

*Acinetobacter baumannii* is a major pathogen known to cause a wide range of serious nosocomial infections presenting as pneumonia, bacteremia, urinary tract infections, meningitis, and surgical wound infections [1]. As a member of the ESKAPE group, *A*. *baumannii* infections are characterized by increasing resistance to commonly used antibiotics, including the carbapenems [2]. Given their high degree of resistance, these isolates are classified as “priority one” pathogens by the World Health Organization, making them a priority for the development of novel or more effective antibiotic treatments [3].

Antibiotic heteroresistance is a term used to describe microbial populations presenting with a smaller antibiotic-resistant subpopulation within a larger single antibiotic-susceptible bacterial isolate [4]. Although more evidence is still needed to understand the development of these populations, it is widely accepted that heteroresistance is a common mechanism facilitating treatment failure due to the selection of these resistant subpopulations after antibiotic treatment [5]. Recent studies have increasingly reported that heteroresistance to diverse antibiotics is frequently detected in *A. baumannii* clinical isolates [6], with many of these studies describing a high degree of colistin heteroresistance among these isolates [7]. In addition, we recently demonstrated that there is also a high proportion of tigecycline heteroresistance in *A. baumannii* clinical isolates [8].

Although tigecycline and colistin are considered last-resort antibiotics for treating multidrug-resistant *A. baumannii* infections [9], their rates of resistance remain of critical interest to the field. In addition, although multiple heteroresistance to tigecycline and colistin has not been previously investigated in *A. baumannii*, the presence of carbapenem-resistant *Enterobacteriaceae* clinical isolates exhibiting heteroresistance to multiple antibiotics has been previously reported [10, 11]. Therefore, it is expected that single antibiotic therapies using tigecycline or colistin are unlikely to be effective against infections by heteroresistant isolates.

In the current study, we report the common presence of multiple heteroresistance exhibiting resistance to both tigecycline and colistin in *A. baumannii* clinical isolates. Our evaluations also revealed that these resistant subpopulations are completely independent and that the combined application of both tigecycline and colistin would be effective during the eradication of multi-antimicrobial heteroresistant *A. baumannii* isolates.

## Materials and methods

### Bacterial isolates and antibiotic susceptibility testing

Eight clinical *A. baumannii* isolates known for their tigecycline heteroresistance in our previous study [8], were used in this study. Tigecycline-resistant subpopulations (FA#-TIG-RP or F-#-TIG-RP) were investigated in our previous study [8] and colistin-resistant subpopulations (FA#-COL-RP or F-#-COL-RP) were obtained from the seven *A. baumannii* isolates (Table 1) after population analysis profiling (PAP). Only FA154-COL-RP could not be available as original isolate FA154 was shown to be resistant to colistin. Genotyping was then performed on each of the parental isolates and their tigecycline- and colistin-resistant subpopulations using multilocus sequence typing (MLST) based on Oxford database [12].

**Table 1:** Genotypes and minimal inhibitory concentrations (MICs) for tigecycline and colistin in eight *A. baumannii* parental isolates and their respective tigecycline- (TIG-RP) and colistin-resistant subpopulations (COL-RP)

Isolate number	Sequence type	MIC (mg/L)					
		Parental		TIG-RP		COL-RP	
		TIG	COL	TIG	COL	TIG	COL
FA56	191	2	2	16	2	2	> 64
FA83	191	2	2	16	2	2	> 64
FA154	191	2	> 64	16	2	NA	NA
FA1318	357	2	2	16	2	2	> 64
FA1323	357	2	2	16	2	2	> 64
F-1757	357	2	2	16	2	2	> 64
F-2420	357	2	2	16	2	2	> 64
FA72	191	4	2	32	2	2	> 64

MIC, minimal inhibitory concentration; TIG, tigecycline; COL, colistin; RP, resistant subpopulation; NA, not applicable (because the parental isolate is colistin-resistant)

We also measured the minimal inhibitory concentrations (MICs) for clinical *A. baumannii* isolates and their tigecycline- and colistin-resistant subpopulations using the standard broth microdilution method, as outlined in the guidelines provided by the Clinical and Laboratory Standards Institute (CLSI) [13]. *Escherichia coli* strain ATCC 25922 was used as a reference for MIC quality control, and the MIC breakpoint for colistin was classified as described by the CLSI; resistant, ≥ 4 mg/L. However, we applied the latest FDA-Identified Interpretive Criteria for *Enterobacteriaceae* when evaluating the tigecycline MIC breakpoints as there is no established breakpoint for tigecycline in *Acinetobacter* spp. [14]; an MIC of ≤ 2 mg/L as susceptible, 4 mg/L as intermediate, and ≥ 8 mg/L as resistant.

### Population analysis profiling (PAP)

Colistin heteroresistance of the seven previously identified tigecycline-heteroresistant and colistin-susceptible isolates was evaluated using PAP. The PAP was conducted as previously described with some modifications [15]. Briefly, overnight cultures of each isolate grown at 37 °C and 220 rpm were serially diluted tenfold in phosphate buffered saline and then spotted as 10 μL suspensions onto Mueller–Hinton (MH) II agar plates with varying concentrations of colistin, with these plates creating a twofold gradient between 1 to 16 mg/L. These plates were then incubated at 37 °C for 24 h and their CFU/mL were then calculated. The proportion of colistin-resistant subpopulations were measured based on the number of surviving cells on any MH agar containing more than 4 mg/L colistin. Antibiotic heteroresistance was defined as the occurrence of resistant subpopulations at eightfold or greater MIC, when compared to their respective parental strain at a frequency of 10^–7^ to 10^–6^ [4]. Colistin-resistant subpopulations were then isolated from these colonies, grown on agar plates with the highest concentrations of colistin evaluated in the PAP and stored as frozen stock.

We also performed PAP using two antibiotics (dual PAP) using a similar approach to that described using plates supplemented with both tigecycline and colistin on the same concentration gradient as described in the single PAP experiments. Viable cells at each concentration were then calculated using the spotting test method.

### Genetic evaluations

Genetic alterations within the coding genes of the PmrAB two-component regulatory system, *pmrAB* and their effector protein-coding gene, *pmrC,* within the colistin-resistant subpopulations were identified by PCR and sequencing (Additional file 1: Table S1). Genomic DNA was extracted from each isolate using a G-spin™ Genomic DNA Extraction kit for bacteria (iNtRON Biotechnology, Seongnam, Korea); any and all mutations in these colistin-resistant subpopulations were analyzed based on their respective parental strains using SnapGene version 4.1.9 (GSL Biotech LLC, Greater Chicago Area, Great Lakes, Midwestern US) and described as amino acid alterations.

### Gene expression analysis

The relative mRNA expression levels of *pmrB* from both the parental isolates and colistin-resistant subpopulations were compared using quantitative reverse transcription polymerase chain reaction (qRT-PCR). Total RNA was extracted from mid-log cultures of each strain using the RNeasy Mini kit (Qiagen, Hilden, Germany) and cDNA was synthesized using HiSenScript™ RH[-] RT PreMix kit (iNtRON Biotechnology, Seongnam, Korea). The qRT-PCR was then carried out using TB Green Premix Ex Taq™ (TaKaRa, Kyoto, Japan) and a QuantStudio™ 7 Flex Real-Time PCR System (Thermo Fisher Scientific, Waltham, Massachusetts, USA). The relative mRNA expression levels of each of the target genes were normalized to the expression level of housekeeping gene *rpoB* using the ΔΔC_T_ method. All evaluations were completed in triplicate.

### In vitro time-killing assay

Next, we investigated the efficacy of combined antibiotic treatment using an in vitro time-killing assay based on the protocol outlined in the CLSI recommendations [16]. Briefly, overnight cultures of each of the seven multiple heteroresistant isolates (F-1757 and F-2420) were diluted 1:100 in MH II broth and exposed to 2 × MICs of tigecycline, colistin, and a combination of the two antibiotics. Cultures were incubated at 37 °C with shaking at 220 rpm for 24 h and viable cell counts were determined at 0, 3, 6, 9, 12, and 24 h using the spotting test as described above.

In addition, the other five multiple heteroresistant *A. baumannii* isolates were also evaluated using an in vitro killing efficacy assay which combined these antibiotics and exposed cells to 2 × MICs. Surviving cells were enumerated only after 24 h of incubation by spotting test using MH II agar plates.

### Statistical analysis

Statistical analyses were completed using a Student’s *t*-test as administered by Prism version 8.3.0, software for Windows (GraphPad Software), and significance was set at *p* < 0.05 (*p < 0.05; **p < 0.01; ***p < 0.001).

## Results

### Identification of colistin heteroresistance among tigecycline-heteroresistant *A. baumannii* isolates

**Fig. 1:**
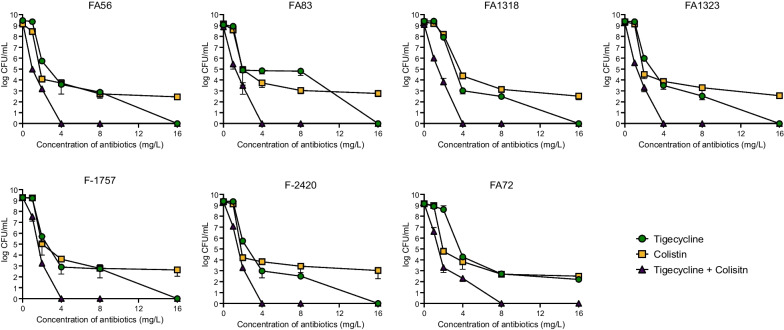
Population analysis profiling (PAP). Colistin heteroresistance in seven tigecycline-heteroresistant, colistin-susceptible *A. baumannii* isolates were identified using PAP analysis using single regimen. The results of dual PAP using tigecycline and colistin in multiple heteroresistant *A. baumannii* isolates did not exhibit heteroresistance phenotype

Of the eight tigecycline-heteroresistant *A. baumannii* isolates, seven were susceptible to colistin (Table 1). These seven isolates were then subjected to PAP using colistin which revealed that all of these colistin-susceptible isolates were also heteroresistant to this antibiotic (Fig. 1). Thus, all seven of these *A. baumannii* isolates were susceptible to both tigecycline and colistin and simultaneously heteroresistant to both antibiotics, defining this characteristic as multiple heteroresistance. In addition, while the proportion of tigecycline-resistant subpopulations in these seven tigecycline-susceptible isolates ranged from 1.2 × 10^–7^ to 5.2 × 10^–5^ [8], the colistin-resistant subpopulations in the same isolates ranged from 2.3 × 10^–6^ to 1.2 × 10^–5^.

Colistin-resistant subpopulations obtained during PAP exhibited a very high level of colistin resistance, exhibiting a more than 32-fold increase in MIC when compared to their respective parental strain (Table 1). In addition, all tigecycline-resistant subpopulations were susceptible to colistin, and all colistin-resistant subpopulations were susceptible to tigecycline (Table 1). Finally, MLST analysis revealed that all of the sequence types (STs) for all of the resistant subpopulations were consistent with the STs of their respective parental strain (Table 1).

### Profile of the antibiotic resistance mechanisms in the colistin-resistant subpopulations

**Fig. 2:**
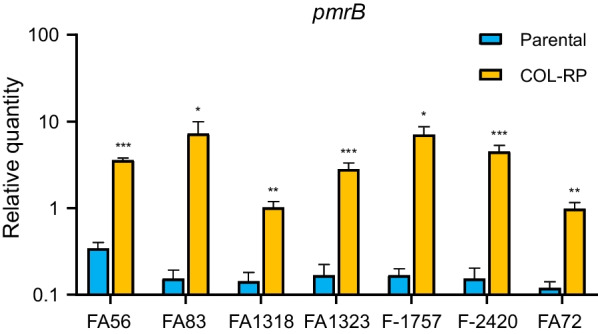
Relative mRNA expression levels of *pmrB* gene in both the parental isolates and their colistin-resistant subpopulations (COL-RP). Expression was measured as a relative quantity by quantitative real-time polymerase chain reaction (qRT-PCR) using reference gene *rpoB*. *p < 0.05; **p < 0.01; ***p < 0.001

A total of six of the colistin-resistant subpopulations exhibited amino acid alterations in PmrB; three (G260D, F222Y, and R263H) in histidine kinas A (HisKA) domain, two (E301K and G315C) in nonfunctional domain, and one (L208F) in HAMP domain (Table 2). One amino acid was found in receiver domain of PmrA in FA1318-COL-RP. Amino acid substitutions in HisKA and HAMP domains of PmrB and receiver domain of PmrA have been reported to be responsible for increase of colistin MIC associated in *A. baumannii* [17–19]. Different amino acid alterations in the seven colistin-resistant subpopulations suggest that each developed independently. None of these strains displayed changes in the PmrC. qRT-PCR-based evaluation of *pmrB* revealed its significant upregulation in all of the resistant isolates when compared to their parental strain (Fig. 2). This suggests that the colistin resistance described in our heteroresistant isolates was mediated by an upregulation of the PmrAB two-component regulatory system which was facilitated by several well-known amino acid alterations [20, 21].

**Table 2:** Summary of the amino acid alterations in the PmrAB two-component regulatory system in colistin-resistant subpopulations (COL-RP)

Isolate number	Colistin MIC (mg/L)	Amino acid alterations		
		PmrA	PmrB	PmrC
FA56-COL-RP	> 64	–	E301K^a^	–
FA83-COL-RP	> 64	–	G260D^b^	–
FA1318-COL-RP	> 64	D82N	–	–
FA1323-COL-RP	> 64	–	F222Y^b^	–
F-1757-COL-RP	> 64	–	R263H^b^	–
F-2420-COL-RP	> 64	–	G315C^a^	–
FA72-COL-RP	> 64	–	L208F^c^	–

^a^Amino acid alternations in nonfunctional domain of PmrB

^b^Amino acid alterations in histidine kinase A (HisKA) domain of PmrB

^c^Amino acid alteration in HAMP domain of PmrB

### Efficacy of combined antibiotic treatment in multiple heteroresistant isolates

We further performed the dual PAP against all seven multiple heteroresistant isolates as explained in Fig. 1. While multiple heteroresistant isolates showed heteroresistant phenotypes for tigecycline and colistin, they did not exhibit a heteroresistance phenotype when tigecycline and colistin were combined, as evidenced by a lack of any cells in media enriched with more than 4 mg/L of both antibiotic during dual PAP.

**Fig. 3:**
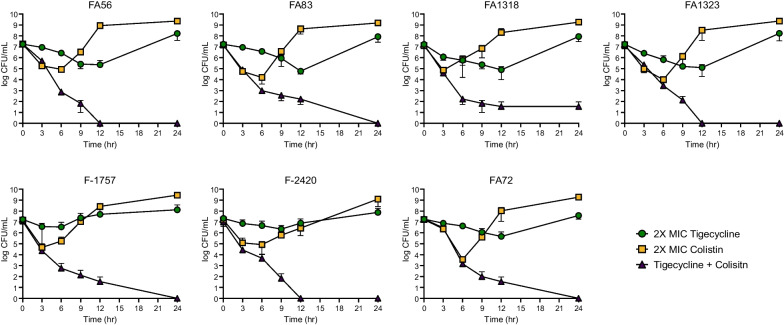
Survival analysis. The results of the in vitro time-killing assay of both monotherapy and combination treatment of tigecycline and colistin in multiple heteroresistant *A. baumannii* isolates. Both tigecycline and colistin were 2 × MIC (4 or 8 mg/L)

In vitro time-killing assays also demonstrated the effectiveness of this combined treatment in multiple heteroresistant isolates (Fig. 3). Despite treatment at 2 × MICs, tigecycline or colistin alone failed to inhibit the growth of either resistant subpopulation. However, combined administration of both tigecycline and colistin eliminated any surviving cells, inhibiting the regrowth of either resistant subpopulation.

## Discussion

Previously, we reported the high prevalence of tigecycline-heteroresistant *A. baumannii* isolates and revealed that this heteroresistance facilitates increased treatment failure via the selection of resistant populations upon exposure to a moderate concentration of tigecycline [8]. In addition, previous our evaluations suggested that this resistance was largely facilitated by the upregulation of the AdeABC efflux pumps following an IS*Aba1* insertion into *adeS* [8]. Herein, we determined if these tigecycline-heteroresistant isolates were also heteroresistant to colistin, another last line antibiotic used in the treatment of *A. baumannii* infections.

Our investigations revealed that of the eight tigecycline-heteroresistant *A. baumannii* isolates identified in our previous study, only one was colistin-resistant. The other seven isolates presented with a colistin heteroresistance phenotype when evaluated by PAP. This finding implies that there is a high prevalence of multiple heteroresistance to tigecycline and colistin in *A. baumannii*, which is similar to the recent findings described for *Klebsiella pneumoniae* [11]. In these evaluations several tigecycline- and polymyxin B-susceptible *K. pneumoniae* isolates from China were shown to be heteroresistant to both antibiotics, with this resistance appearing in nearly 80% of their isolates. In addition, when these observations are combined with our results, these findings suggest that multiple heteroresistance may be reasonably prevalent in several gram-negative pathogens.

The results of the in vitro antibiotic susceptibility testing of the resistant colonies obtained during our PAP demonstrated that all of the tigecycline-resistant subpopulations were susceptible to colistin, and all of the colistin-resistant subpopulations were susceptible to tigecycline. Thus, no two subpopulations were shown to be simultaneously resistant to both antibiotics suggesting that each resistant subpopulation exists as a distinct event.

The independent existence of tigecycline- and colistin-heteroresistant subpopulations suggests an experimental basis for evaluating the efficacy of combination antimicrobial treatment. As expected, single use of tigecycline or colistin did not reduce the growth of the multiple heteroresistant isolates due to the selection of resistant subpopulations. However, a combination of tigecycline and colistin eradicated almost all of the *A. baumannii* isolates when administered at 2 × MICs of both antibiotics. These evaluations revealed total eradication at 4 mg/L for all but one isolate (tigecycline for FA72, 8 mg/L). Dual PAP confirmed the absence of a combined heteroresistance pattern, suggesting that effective eradication of multiple heteroresistant *A. baumannii* isolates can be achieved using a combination of therapies as the tigecycline and colistin heteroresistance exist in different subpopulations with different resistance patterns within the same isolate.

Our study have some limitations. First is that only in vitro studies were conducted. Second, we did not investigate the distribution of multiple heteroresistant *A. baumannii* strains in the clinical settings. Further studies through systematic collection of clinical isolates and in vivo assays are guaranteed.

## Conclusions

Many studies have shown the limitations of colistin or tigecycline monotherapy and the success of combining these antibiotics [22–25]. However, few studies have elucidated the underlying mechanism. Our study revealed that prevalent heteroresistance is likely a significant factor in the failures of antibiotic monotherapy and suggests that combined antibiotic therapies may facilitate better clinical outcomes via their independent eradication of heteroresistant subpopulations.

## Supplementary Information

**Additional file 1: Table S1.** List of primers used in this study.

## Data Availability

All materials are available by the corresponding author.

## Abbreviations

PAP: Population analysis profiling
TIG: Tigecycline
COL: Colistin
RP: Resistant population
MLST: Multilocus sequence typing
CLSI: Clinical and Laboratory Standards Institute
MIC: Minimum inhibitory concentration
MH: Mueller–Hinton
CFU: Colony-forming unit
qRT-PCR: Quantitative reverse transcription polymerase chanin reaction
ST: Sequence type
