# Supplementary material for: Multiple heteroresistance to tigecycline and colistin in Acinetobacter baumannii isolates and its implications for combined antibiotic treatment
Source: J Biomed Sci. 2023 Jun 7;30:37. doi: 10.1186/s12929-023-00914-6 (PMC10245395; doi:10.1186/s12929-023-00914-6)
Supplement: Supplementary file 1 — Additional file 1: Table S1. List of primers used in this study. [file 12929_2023_914_MOESM1_ESM.pdf]

Table S1. List of primers used in this study

Gene	Primers	Sequence (5′-3′)
PCR and sequencing		
pmrA	pmrA-F	ACTGGACATGTTGCACTCTT
	pmrA-R	TGAAGTGCAACCTTATAAGCAC
pmrB	pmrB-F	ATTCGAACCATCCGAGGACT
	pmrB-R	TGCGAGGAGCACATTTTCTA
pmrC	pmrC-F	CGGTAAGCGTGATACCTTTGA
	pmrC-R	GAGCCAAACCATCTAAACCGT
	pmrC-IF	CCGTGGTCGGTGTTTTACTT
	pmrC-IR	CCTTCGCGGTGACTAGCTAA
qRT-PCR		
pmrB	qpmrB-F	CGGGCAGTATGTTTATTCCG
	qpmrB-R	CGGAATCGCGTTCTTTTAAC
rpoB	qrpoB-F	GCAAGATGGCAAATCACCAA
	qrpoB-R	TTCTAAAGCAGCATTGCCAGAA
